# Supplementary material for: Identification of IMP Dehydrogenase as a Potential Target for Anti-Mpox Virus Agents
Source: Microbiol Spectr. 2023 Jul 6;11(4):e00566-23. doi: 10.1128/spectrum.00566-23 (PMC10434032; doi:10.1128/spectrum.00566-23)
Supplement: Supplemental file 1 — Supplemental material. Download spectrum.00566-23-s0001.pdf, PDF file, 0.6 MB [file spectrum.00566-23-s0001.pdf]

Supporting Information

Identification of inosine monophosphate dehydrogenase as a potential target for anti-mpox virus agents

Takayuki Hishiki[1†], Takeshi Morita[1†], Daisuke Akazawa[1¶], Hirofumi Ohashi[1¶], Eun-Sil Park[2], Michiyo Kataoka[3], Junki Mifune[1], Kaho Shionoya[4,5], Kana Tsuchimoto[1], Shinjiro Ojima[1], Aa Haeruman Azam[1], Shogo Nakajima[4], Madoka Kawahara[1,6], Tomoki Yoshikawa[6], Masayuki Shimojima[6], Kotaro Kiga[1], Ken Maeda[2], Tadaki Suzuki[3], Hideki Ebihara[6], Yoshimasa Takahashi[1], Koichi Watashi[1,4,5,7#]

[1]Research Center for Drug and Vaccine Development, National Institute of Infectious Diseases, Tokyo 162-8640, Japan, [2]Department of Veterinary Science, National Institute of Infectious Diseases, Tokyo 162-8640, Japan, [3]Department of Pathology, National Institute of Infectious Diseases, Tokyo 162-8640, Japan, [4]Department of Virology II, National Institute of Infectious Diseases, Tokyo 162-8640, Japan, [5]Department of Applied Biological Science, Tokyo University of Science, Noda 278-8510, Japan, [6]Department of Virology I, National Institute of Infectious Diseases, Tokyo 162-8640, Japan, [7]MIRAI, Japan Science and Technology Agency (JST), Saitama 332-0012, Japan

[†¶]These authors contributed equally to this work.

[#]Corresponding author: Koichi Watashi, Ph.D.
Research Center for Drug and Vaccine Development, National Institute of Infectious Diseases, 1-23-1 Toyama, Shinjuku-ku, Tokyo 162-8640, Japan
Phone: +81-3-5285-1111; E-mail: kwatashi@niid.go.jp

Table of contents

Fig. S1, S2, S3, S4, and S5
Table S1 and S2

Fig. S1

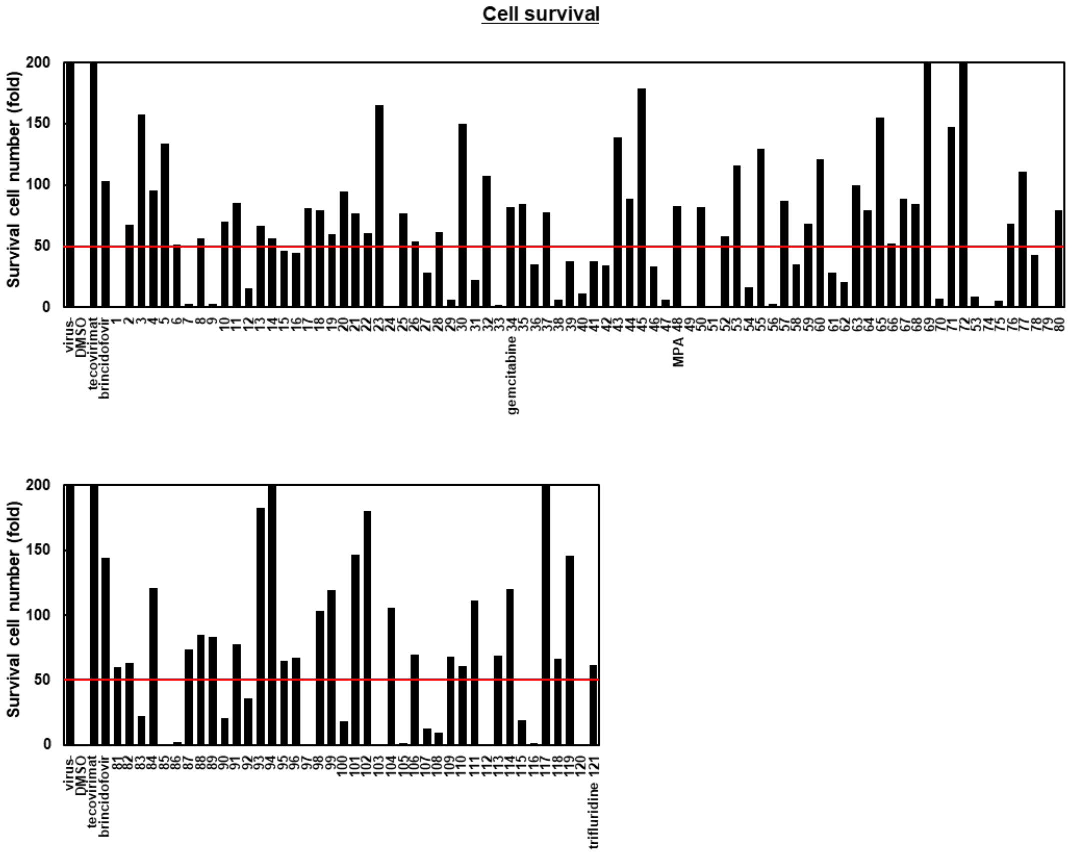

Fig. S1 Primary screening of the compound library in MPXV-infected VeroE6 cells. VeroE6 cells were infected with or without MPXV at an MOI of 0.1 and treated with compounds at 10 μM (2 compounds treated at 2 μM are shown in Table S1) or 0.1% DMSO. Tecovirimat and brincidofovir were used as positive controls. After 72 h of infection, the number of surviving cells was quantified by DAPI staining using a high-content imaging analyzer. The red line shows a 50-fold higher cell survival rate relative to that of the DMSO control in MPXV-infected cells. The numbers (1 to 121) in x-axis corresponds to the compounds listed in Table S1.

Fig. S2

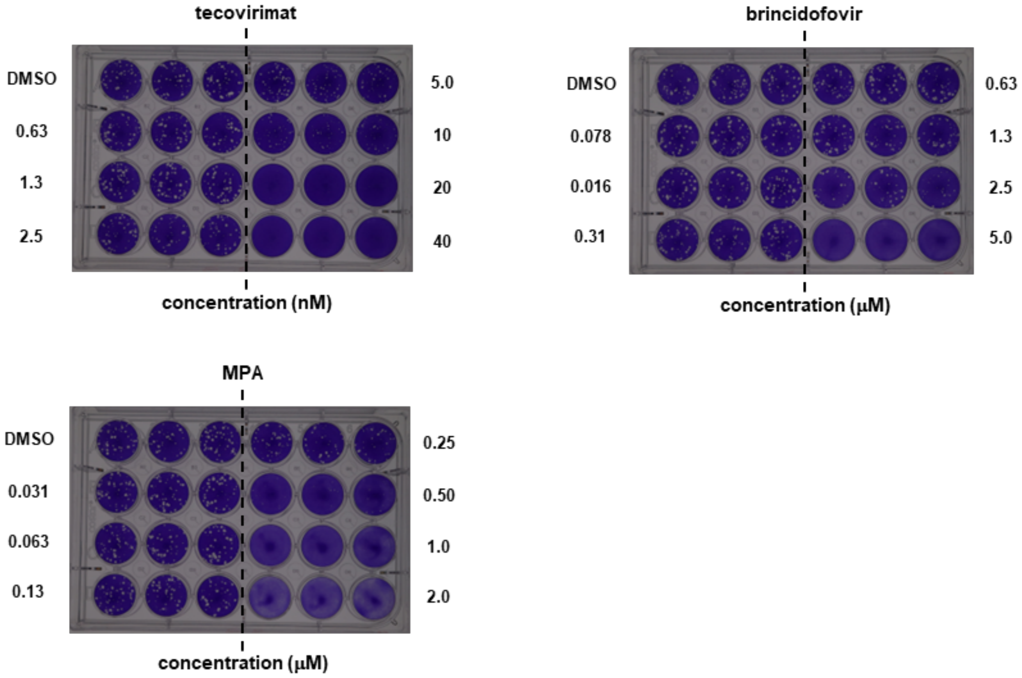

Fig. S2 Evaluation of antiviral activity by plaque reduction assay

VeroE6 cells were seeded at 1 x 10^5 cells/well in a 24-well plate. Cells were infected with MPXV together with the indicated concentrations of compounds. After 72 h of infection, cells were fixed and stained with crystal violet to count the plaque number.

Fig. S3

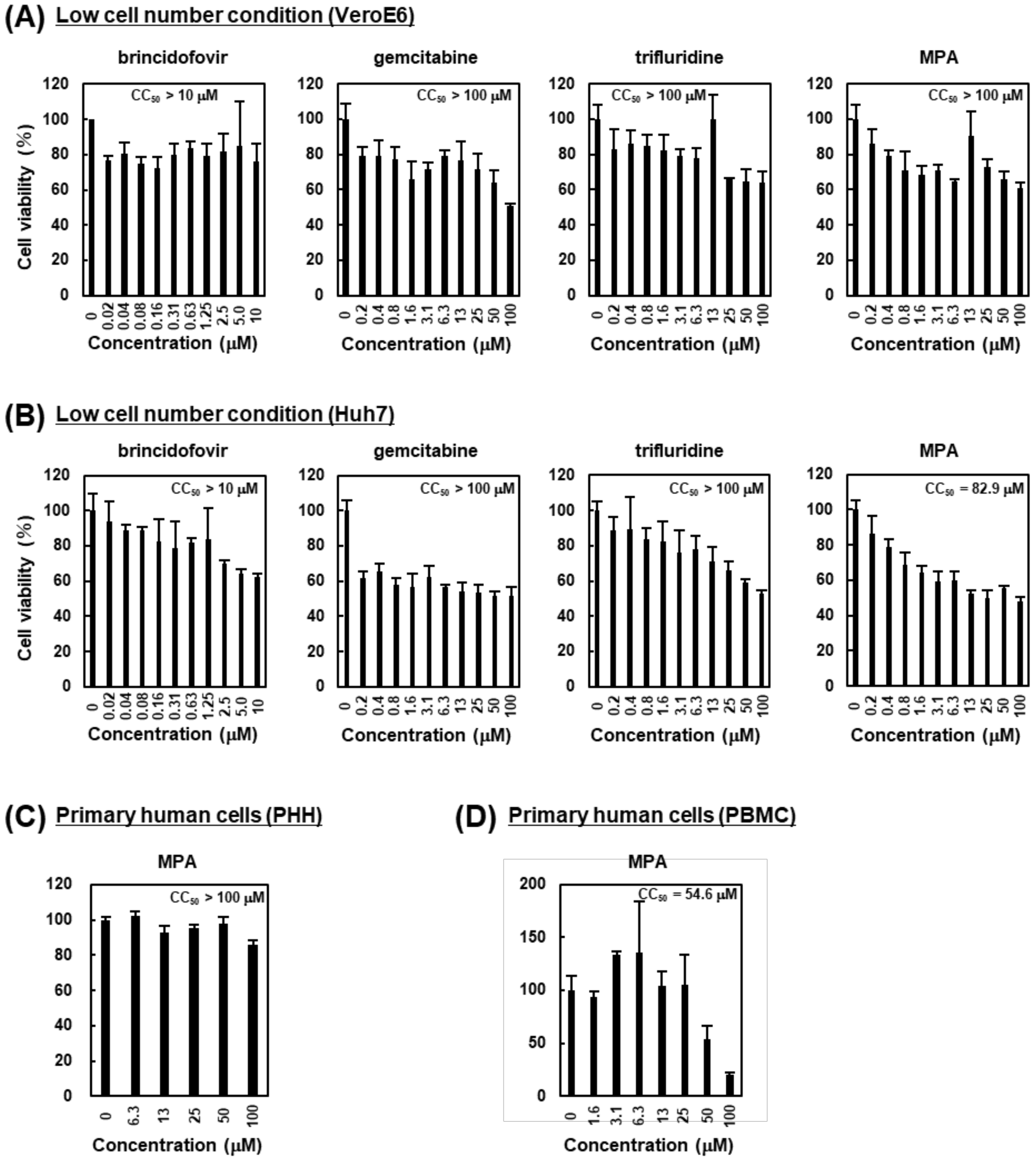

Fig. S3 Cytotoxicity assay in various cells

(A) VeroE6 and (B) Huh7 cells were seeded at 5 x 10^3 cells/well in a 96-well plate as low cell number condition (normally, 2 x 10^4 cells/well). (C) Primary human hepatocytes (PHH) were seeded at 7 x 10^4 cells/well and (D) primary human peripheral blood mononuclear cells (PBMC) were seeded at 1 x 10^5 cells/well in a 96-well plate,

respectively. These cells were incubated with the indicated concentrations of compound for 72 h were subjected to the detection of cell viability. The y-axis shows values relative to that of the DMSO-treated cells as a control.

Fig. S4

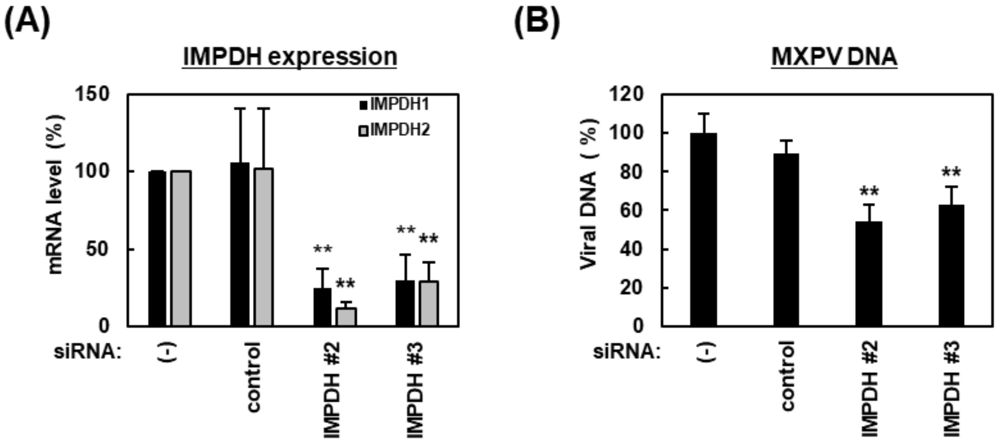

Fig. S4 Suppression of IMPDH expression reduces viral DNA

(A) Huh7 cells were transfected with or without [(-)] siRNA targeting IMPDH (IMPDH) or randomized control siRNA (control). At 48 h post-transfection, intracellular RNA for IMPDH1, IMPDH2, and actin were detected by real-time RT-PCR. The y-axis shows the value realtive to that for the untransfected cells. (B) Intracellular MPXV DNA levels at 72 h post-transfection with siRNA were quantified by real-time PCR and are shown as the percentage relative to that of the untransfected cells. Statistical significance is shown.

Fig. S5

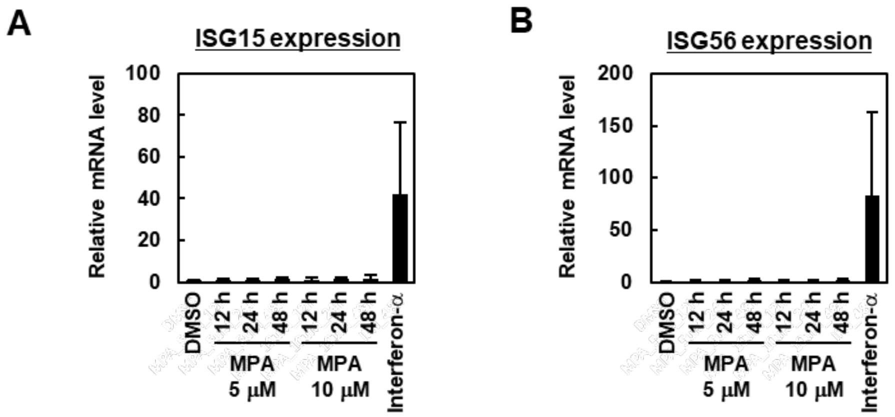

Fig. S5 Expression of interferon-stimulated genes (ISGs) upon MPA treatment. Huh7 cells were incubated with MPA (5 or 10 μM), interferon-α (1000 U/ml), or DMSO (0.1%). After incubation with MPA for 12, 24, and 48 h or with interferon-α or DMSO for 48 h, intracellular RNA was extracted and mRNA expression levels of ISG15 (A) and ISG56 (B) were measured by real-time RT-PCR. The Y-axis shows the value relative to that of DMSO-treated cells as a control.

Table S1 List of drugs in the library

1	ABT-737
2	Linifanib
3	Dovitinib
4	Dasatinib
5	Gefitinib
6	Luminespib
7	MLN8054
8	Cabozantinib
9	Mocetinostat
10	BMS-754807
11	Tanespimycin
12	Delanzomib
13	Ganetespib
14	Onalespib
15	ABT-751
16	BIIB021
17	WZ8040
18	ENMD-2076
19	Cladribine
20	Methotrexate
21	Clofarabine
22	YM201636
23	OSI-930
24	Etoposide
25	KU-0063794
26	Vincristine sulfate
27	BX-912
28	Floxuridine
29	Genistein
30	SP600125
31	HMN-214
32	Fludarabine
33	Selisistat
34	Gemcitabine

35	Adefovir Dipivoxil
36	Azacitidine
37	Cyclocytidine HCl
38	Atorvastatin Calcium
39	Gandotinib
40	Ixazomib
41	Ixazomib Citrate
42	Avasimibe
43	OSI-420
44	UK 383367
45	Apigenin
46	Phloretin
47	Tolbutamide
48	Mycophenolic acid
49	MG-132
50	OSI-027
51	URB597
52	PF-04929113
53	WYE-125132
54	ICG-001
55	Ibrutinib
56	KW-2478
57	Mardepodect
58	KX2-391
59	AMG-900
60	MK-2461
61	Nocodazole
62	RITA
63	Vistusertib
64	Lonafarnib
65	AZD4547
66	TAE226
67	TPCA-1
68	StemRegenin 1
69	Golvatinib

70	ML130
71	WHI-P154
72	CCG 50014
73	Niclosamide
74	Anagrelide HCl
75	Fexofenadine HCl
76	Cabozantinib malate
77	Nifuroxazide
78	PD168393
79	Oprozomib
80	PP1
81	XL888
82	SC144
83	KPT-185
84	SKI II
85	Skepinone-L
86	KPT-276
87	CNX-774
88	NMS-E973
89	Rociletinib
90	TG003
91	PTC-209
92	Sorafenib
93	CGP 57380
94	AR-A014418
95	VER-49009
96	Triapine
97	Afatinib Dimaleate
98	Tenovin-1
99	Tyrphostin AG 1296
100	Butein
101	Ivacaftor
102	Vidarabine
103	Teniposide
104	Cyclosporin A

105	Kaempferol
106	PAC-1
107	Azaguanine-8
108	Bergapten
109	NSC 319726
110	PD153035
111	Miconazole Nitrate
112	Navitoclax
113	Cytarabine
114	Erlotinib HCl (2 μM)
115	Torin 1 (2 μM)
116	Drospirenone
117	Idoxuridine
118	Ciclopirox
119	Econazole nitrate
120	Norethindrone acetate
121	Trifluridine

Compounds were treated at 10 μM, with the exception of 114 and 115, which were treated at 2 μM.

Table S2 Comparison of antiviral activity determined by plaque assay and that by viral DNA quantification

Compound	IC_{50} of plaque	IC_{50} of viral DNA	Significance
tecovirimat (nM)	8.97 ± 1.56	4.60 ± 1.41	N.S.
brincidofovir (μM)	2.58 ± 0.13	2.10 ± 0.69	N.S.
MPA (μM)	0.34 ± 0.09	0.26 ± 0.02	N.S.

Table S2

In comparison of IC_{50} values between viral DNA levels and plaque reduction, the statistical analysis was performed using GraphPad Prism 9 software, and significance was determined using Mann-Whitney U test. N.S.; not significant.
